# In Vitro and In Vivo Models for Analysis of Resistance to Anticancer Molecular Therapies

**DOI:** 10.2174/09298673113209990226

**Published:** 2014-05

**Authors:** Roberta Rosa, Francesca Monteleone, Nicola Zambrano, Roberto Bianco

**Affiliations:** 1 Dipartimento di Medicina Clinica e Chirurgia, 80131 Napoli, Italy; 2 CEINGE Biotecnologie Avanzate, 80145 Napoli, Italy; 3 Associazione Culturale DiSciMuS, 80020 Casavatore (NA), Italy; 4 Dipartimento di Medicina Molecolare e Biotecnologie Mediche, Università di Napoli Federico II, 80131 Napoli, Italy

**Keywords:** Anticancer molecular therapies, tumour, molecular modelling.

## Abstract

The efficacy of classical and molecular therapies in cancer is hampered by the occurrence of primary (intrinsic) and secondary (acquired) refractoriness of tumours to selected therapeutic regimens. Nevertheless, the increased knowledge of the genetic, molecular and metabolic mechanisms underlying cancer results in the generation of a correspondingly increasing number of druggable targets and molecular drugs. Thus, a current challenge in molecular oncology and medicinal chemistry is to cope with the increased need for modelling, both in cellular and animal systems, the genetic assets associated to cancer resistance to drugs. In this review, we summarize the current strategies for generation and analysis of *in vitro* and *in vivo* models, which may reveal useful to extract information on the molecular basis of intrinsic and acquired resistance to anticancer molecular agents.

## 1. INTRODUCTION

Through the use of selective anticancer molecular agents, hitting specific molecular pathways involved in tumorigenesis and/or tumor progression, targeted therapy has changed the approaches to treat cancer during the last 10 years. Still conventional chemotherapy represents the treatment of choice in cancer; however, targeted therapies have become part of the therapeutic strategy for many solid tumors, such as breast, colorectal, lung and renal cancers, as well as for hematologic malignancies such as lymphoma, leukemia and multiple myeloma [1].

The generation and the analysis of cellular and/or animal systems has provided several examples of the usefulness for modeling the genetic defects of cancer in the definition of responsiveness or resistance to targeted therapy. The paradigm of acute promyeolocytic leukemia (APL) is a virtuous proof-of-concept, in which the clear overlap between the human disease and the animal model resulted in the definition of a highly effective therapeutic regimen [2]. The latter is based on the As_2_O_3_ and retinoic acid combination, accounting for almost 100% complete remissions of t(15;17) APL [3]. Such example does indeed underscore the relevance of generating and analyzing the appropriate models in the analysis of drug sensitivities and resistances. Thus, it represents a framework for extension of this concept to additional hematological and solid malignancies. However, the complexity of genetic and epigenetic alterations in cancer invariably highlights tremendously complex *scenarios*; on the other hand, more and more sophisticated molecular tools now allow to systematically dissect the genetic, molecular and functional signatures of cancer. The availability of such information should direct the set-up of appropriate models to recapitulate sensitivity and resistance to targeted molecular therapy in cellular and animal systems. 

The current targeted therapies are mainly based on monoclonal antibodies (mAbs) and small molecule tyrosine kinase inhibitors (TKIs) able to interfere with molecules involved in cancer development and progression. Some patients are intrinsically resistant to these therapies, namely, they do not achieve stable disease or progress within 6 months of treatment. In these cases, administration of a targeted agent may be even detrimental, as patients do not derive any benefit during treatment and may develop side effects deriving from drug toxicity [4]. Consequently, it is important to carefully select patients who could potentially benefit from the treatment, but also to investigate the molecular basis of unresponsiveness. Thus, modeling the genetic complexity of tumors in cellular and animal models may, on one hand, complement the retrospective studies to facilitate prediction of resistance; on the other hand, these models are widely used to perform systematic, molecular and functional studies, to analyze the mechanisms underlying resistance, and propose/test strategies to circumvent resistances.

Resistance to targeted therapy typically accounts on three main cellular mechanisms including 1) the occurrence of mutations in genes encoding target molecules, that abrogate molecular drug binding; 2) the amplification of the target; 3) the up-regulation of alternative proliferation/survival pathways. Among genetic alterations, the Epidermal Growth Factor Receptor (EGFR) variant III (EGFRvIII) is the result of an in-frame deletion from exons 2 through 7 in the extracellular domain, that prevents EGFRvIII from binding EGF and other ligands. The constitutive signaling by EGFRvIII and the resistance to EGFR-targeted therapy are thought to be dependent from structural changes in the EGFR protein that, in turn, could affect the intracellular domain conformation and the ATP pocket. This ligand-independent mutant protein is constitutively activated and has been observed in up to 40% of glioblastomas and in other human malignancies [5]. Loss of PTEN, a tumor-suppressor protein that is commonly lost in gliobastoma and that inhibits the phosphoinositide 3-kinase (PI3K) signaling pathway, may also promote resistance to EGFR or HER2 inhibitors [6, 7]. On the other hand, host-related mechanisms could also be responsible for intrinsic cancer cell resistance, such as a defective immune system-mediated function, a rapid inactivating metabolism or a poor absorption. For example, impairment of antibody- dependent cell-mediated cytotoxicity (ADCC), an important mechanism of action of several mAbs *in vivo*, including the anti-EGFR mAb cetuximab and the anti-HER2 mAb trastuzumab, could result in an intrinsic host-related resistance to treatment with these agents [8].

Secondary or acquired resistance to targeted agents typically arises after prolonged treatments in cancer patients. In fact, even if an initial response is obtained, the vast majority of tumors subsequently become refractory, and disease progression eventually occurs. Several molecular mechanisms have been suggested to contribute to the resistant phenotype. Among them, selection of tumor cells with specific mutations making drugs unable to bind to their specific target. For tyrosine kinase inhibitors (TKIs) [9], mutations within the ATP pocket of the kinase domain may affect the interaction of the drug with its target: for example, the single amino acid mutation tyrosine/methionine (T790M) in the ATP binding pocket of the EGFR mediates secondary resistance to the EGFR TKIs erlotinib and gefitinib in non-small cell lung cancer (NSCLC) [10, 11]. The T790M mutation in EGFR is structurally analogous to drug resistance mutations in other kinases such as BCR-ABL (T315I), c-KIT (T670I) and platelet-derived growth factor receptor-α (PDGFRα) (T674I) [12]. Furthermore, acquired resistance may be mediated by activation of redundant signaling pathways able to bypass the blockade of single proteins involved in cancer cells proliferation, survival, or migration. Having again the EGFR inhibitors as a paradigm, this is the case of K-Ras mutations leading to constitutive activation of the Ras/MAPK signaling pathway and mediating both intrinsic or acquired resistance to the anti-EGFR mAb cetuximab in colorectal cancers [13]. Similarly, amplification of Met causes gefitinib resistance by driving HER3-dependent activation of the PI3K/Akt pathway [14]. In this complex scenario, cellular and animal models, in combination with molecular studies, may be of help in the development of novel inhibitors against drug-resistant tumors [15].

Unconventional mechanisms mediate resistance to another class of molecular drugs, the anti-angiogenic agents [16, 17]. Evasion of anti-angiogenic therapy is indeed largely indirect. Alternative ways to sustain tumor growth are activated through different mechanisms: up-regulation of alternative pro-angiogenic signaling pathways; recruitment of bone marrow-derived pro-angiogenic cells; increased pericyte coverage of the tumor vasculature; enhancement of invasion and metastasis to provide access to normal tissue vasculature without obligate neovascularization [18]. 

Each of the described mechanisms only partially justifies the lack of durable responses to targeted drugs observed in cancer patients. Therefore, the search for further determinants of resistance may help to better select those patients who are potentially long-term responders to specific classes of molecular agents. Moreover, a better understanding of the molecular mechanisms driving the development of secondary resistance may open the path to develop novel therapeutic strategies based on combinations of conventional and targeted agents for resistant cancers. 

In this review, we will focus on the current strategies to generate and analyze cellular and animal models, to extract information on the molecular basis of resistance to anticancer molecular agents, in both paradigms of intrinsic (Table **1**) and acquired resistance (Table **2**).

## 2. INTRINSIC RESISTANCE 

Several preclinical models, useful to clarify mechanisms of intrinsic resistance to molecular agents, have been selected in the last years. Therefore, in the first part of this review, we will focus on these *in vitro* and *in vivo* models (Table **1**). 

### 2.1. Cellular Models of Intrinsic Resistance 

The study of the molecular basis of intrinsic resistance to targeted agents takes advantages from mainly two types of *in vitro* models: human immortalized cancer cell lines, derived from cancer patients showing primary resistance, and primary cultures of cells often directly obtained at the time of diagnosis from human cancers, whose sensitivity or resistance to a specific molecular anticancer drug has to be later evaluated. 

As many cell lines are available for each cancer type, carrying different genetic alterations and showing different degrees of sensitivity to targeted therapies, several bioinformatics tools have been developed to assist researchers in the preliminary step of choosing the most suitable *in vitro* models to investigate mechanisms of intrinsic resistance to anticancer molecular drugs. Two of them are the Genomics of Drug Sensitivity in Cancer (GDSC) database and the Cancer Cell Line Encyclopedia (CCLE). The GDSC database (www.cancerRxgene.org) is the largest public resource for information on drug sensitivity in cancer cells and molecular markers of drug response; it integrates cell lines drug sensitivity data with information on somatic mutations, amplifications and deletions, tissue type and transcriptional data. This body of information is obtained from the Catalogue of Somatic Mutations in Cancer (COSMIC) database [19], a resource for annotation of somatic mutations in cancer [20]. Cancer cell lines drug sensitivity data are generated from screening of a panel of several hundred cancer cell lines with 130 drugs under clinical and preclinical investigation, performed within the Cancer Genome Project at the Wellcome Trust Sanger Institute (WTSI) and the Center for Molecular Therapeutics at Massachusetts General Hospital [21]. CCLE (www.broadinstitute.org/ccle) is a compilation of gene expression, chromosomal copy number and massively parallel sequencing data from 947 human cancer cell lines. In 479 cell lines, these data are coupled with pharmacological profiles for 24 anticancer drugs, so allowing identification of genetic, lineage and gene-expression-based predictors of drug sensitivity [22]. 

Reflecting the large number of cell lines available and the ease with which the latter are cultured and manipulated, there are numerous examples of *in vitro* models used to investigate mechanisms of intrinsic resistance to anticancer molecular agents. In the breast cancer setting, different models to study the clinically relevant resistance to the anti-HER2 mAb trastuzumab are available. For example, JIMT-1 is a trastuzumab-resistant cell line, established from a breast cancer patient showing HER2 gene amplification and primary resistance to trastuzumab [23]. Nagy *et al.* have shown that the trastuzumab binding epitope of HER2 in JIMT-1 was masked by the membrane-associated glycoprotein MUC4, leading to diminished binding of trastuzumab and consequently to intrinsic resistance to treatment [24]. Otherwise, it has been demonstrated that resistance to trastuzumab could be related to cleavage of the full-length 185 kDa HER2 protein by matrix metalloproteases. This event produces a 110 kDa extracellular domain (ECD), which is released into cell culture media or circulates in serum *in vivo*, and a 95 kDa NH2-terminal truncated membrane-associated fragment with increased kinase activity, defined as p95HER2 [25]. The KPL-4 cell line, isolated from the malignant pleural effusion of a breast cancer patient with an inflammatory skin metastasis and resistant to trastuzumab in female athymic nude mice [26], represents another model of intrinsic resistance to trastuzumab, due to p95HER2 fragment overexpression. Important findings have suggested a strong relationship between resistance to EGFR TKIs and the absence of activating mutations in the intracellular domain of the receptor [27]. These EGFR kinase mutations enhance ligand-dependent activation of EGFR, and simultaneously increase sensitivity to TKIs [28]. Approximately 90% of these EGFR gene mutations affect the small region of the gene within the exons (18–24) that code for the TK domain. The most common mutations are an in-frame deletion in exon 19 around codons 746–750 (45%–50% of all somatic EGFR mutations) and a missense mutation leading to leucine to arginine substitution at codon 858 (L858R) in exon 21 (35%–45% of mutations) [29]. Several NSCLC cell lines lacking the above cited mutations are considered valuable models to study intrinsic resistance to the EGFR TKIs gefitinib and erlotinib, such as A549, H460, H1299 and GLC-82. Some of these cell lines also show mutations in the genes coding for the downstream transducers K-Ras or PI3K [30-32]. In the colorectal cancer setting, preclinical *in vitro* models of resistance to the anti-EGFR mAbs cetuximab and panitumumab include cell lines showing mutations of the K-Ras gene, most frequently in codon 12 of exon 2, such as SW480, LS174T, HCT116, LoVo cells. These mutations produce a single amino acid change resulting in mutant Ras proteins that are insensitive to GAP function and constitutively active, with consequent activation of the Ras/MAPK signaling [33]. Furthermore, several colorectal cancer cell lines (VAC0432, SNU-C5, HT29, KM20, WiDr) are considered valuable models of resistance to the B- Raf (V600E) inhibitor vemurafenib [34] because of the high levels of EGFR expression. Mechanistically, B-Raf (V600E) inhibition causes a rapid feedback activation of EGFR, which supports continued proliferation in the presence of vemurafenib [35]. Finally, colorectal cancer cells with no mutations in the B-Raf or K-Ras genes (HCA7, CaCo2, COLO320DM) show intrinsic resistance to the highly potent, selective and ATP uncompetitive inhibitor of MEK1/2 kinases selumetinib [36]. Among the cells with high ERK1/2 activity (whether mutant for B-Raf or K-Ras), intrinsic resistance to selumetinib seems to be related to high PI3K-dependent signaling (RKO, CO115, DLD-1, SW837 cells) [37]. In the field of hematologic malignancies, the event of intrinsic resistance to the highly selective, reversible inhibitor of the 26S proteasome bortezomib appears to be clinically relevant in mantle cell lymphoma (MCL). MINO and REC1 MCL cell lines have been used to characterize mechanisms of bortezomib resistance. In these cells, expression of cell-surface plasmacytic differentiation markers CD38 and CD138, and up-regulation of the transcription factor IRF4 have been found; these *in vitro* models helped to confirm that, in some MCL patients, resistance to bortezomib may be mediated through a partial plasmacytic differentiation to tolerate the accumulation of intracellular proteins during proteasome inhibition.

### 2.2. Animal Models of Intrinsic Resistance

*In vivo* models provide the native microenvironment in which tumors reside, so they are more advantageous than *in vitro* ones. The most frequently used *in vivo* models are the human tumor xenografts obtained inoculating both immortalized human cancer cells or small fragments from cancer specimens showing intrinsic resistance to anticancer targeted agents. In order to avoid the reject of the implanted human cancer cells, mice used for xenografts should be immunocompromised, such as: 

Athymic Nude Mice (Balb/c, CD-1, Nu/Nu): These animals lack a thymus and are unable to produce T cells. In addition to the *nude* gene, they can exhibit additional mutations such as the *xid* mutation, affecting the maturation of T-independent B lymphocytes, or the *beige* mutation, resulting in defective natural killer (NK) cells;

SCID Mice: These animals show a severe combined immunodeficiency affecting both B and T lymphocytes. They have normal NK cells, macrophages, and granulocytes, unless they exhibit also the *beige* mutation resulting in defective NK cells [38]. 

One advantage of tumor xenografts is that the starting material is usually derived from advanced cancers or metastasis; the cells presumably represent real human tumors replete with genetic complexity. However, tumor-derived cell lines may not completely recapitulate intra-tumor heterogeneity, because the cells that grow may represent only a subpopulation of the whole tumor; moreover, tumor growth occurs in a host with an impaired immune system. Syngeneic models of cancer, where murine cancer cells are implanted in mice, can be especially useful when the molecule under evaluation interacts with the host immune system. In this situation, using athymic or SCID mice may prevent a drug from working properly, while using an immunocompetent syngeneic model where animal has a complete and intact immune system can allow for a more accurate evaluation of the drug activity. Furthermore, genetically engineered mouse models (GEMMs), mice in which an oncogene could be turned on or a tumor suppressor gene can be turned off in specific tissues at specific times (by addition or removal of an inducer in the animal diet), may be also useful. In GEMMs, tumors arise *in situ* where immune function, angiogenesis, and inflammatory processes can all interact normally with the developing tumor. Thus, GEMMs allow for the analysis of tumors as they develop through defined stages of tumorigenesis, facilitating studies of the biology of the tumors early and late in the process [39]. However, GEMMs are more expensive and difficult to use for a large-scale screening of drug candidates, because they develop tumors with long latency and variable penetrance [40].

The use of *in vivo* models is particularly necessary when biological processes such as angiogenesis and conditions such as hypoxia have to be investigated, for example in the case of anti-angiogenic agents. The role of myeloid cells in refractoriness to anti-Vascular Endothelial Growth Factor (VEGF) therapy have been elucidated through syngeneic implantation of murine tumor cell lines in immunocompetent C57BL/6 or immunocompromised XID mice. Since the growth of LLC and EL4 tumors was only modestly and transiently inhibited by treatment with an anti-VEGF mAb, they were established as a model of refractoriness to VEGF targeted agents. Such resistance seemed to be associated with infiltration of the tumor tissue by CD11b+Gr1+ myeloid cells including neutrophils, macrophages, and myeloid-derived suppressor cells. Consistently, gene expression analysis in CD11b+Gr1+ cells isolated from the bone marrow of mice bearing refractory tumors revealed higher expression of a distinct set of genes known to be implicated in active mobilization and recruitment of myeloid cells [41]. Particularly, resistance to anti-VEGF agents may be, at least in part, driven by the secreted protein Bv8, which is up-regulated by the important myeloid growth factor granulocyte colony-stimulating factor (G-CSF) [42].

A transgenic mouse model of pancreatic neuroendocrine carcinogenesis (RIP1-Tag2), in which expression of SV40 T antigens under the control of the rat insulin gene promoter (RIP) invokes a multistage pathway to pancreatic neuroendocrine tumors (PNET) of the islet β cells, engages the IGF signaling pathway, as revealed by its dependence on IGF-II and by accelerated malignant progression upon IGF-1R overexpression. Surprisingly, this model has been described as intrinsically resistant to treatment with an anti-Insulin-like Growth Factor 1 Receptor (IGF-1R) mAb, because of the high levels of insulin receptor (IR) activation [43]. A very interesting model to study intrinsic resistance to mAbs due to host-related mechanisms such as lack of ADCC is represented by a mouse knocked out for the common γ chain of the FC receptor (FCγR), found on NK cells and responsible for ADCC response. In FCγR -/- nude mice, reduced anti-tumor effects of human IgG1 backbone antibodies such as cetuximab, trastuzumab and rituximab were observed compared to FCγR +/+ mice [44].

Very recently, sophisticated GEMM models of lung cancer were used to evaluate responsiveness to combined therapy of docetaxel and the MEK inhibitor selumetinib (AZD6244), according to a co-clinical trial effort [45]. Co-clinical trials aim to anticipate, in suitable GEMMs, the results of concomitant human clinical trials, as well as to provide a rationale in the elaboration of clinically relevant hypotheses, useful for design of corresponding studies in human cancer [2, 45]. In this model, the KRAS G12D activating mutation was associated either to TP53 or LKB1 loss in lung epithelium, to parallel selected human NSCLC cases harboring both oncogene activation (KRAS) and oncosuppressor (TP53 or LKB1) loss. The latter were obtained via nasal instillation of adenoviruses expressing CRE recombinase in the appropriate genetic models. The study showed that the combination therapy with selumetinib was more effective than docetaxel alone in K-Ras and K-Ras/p53 mice, while K-Ras/Lkb1 mice where showing primary resistance to the combination therapy. Finally, stratification of both mice and patients according to LKB1 presence or loss in the KRAS mutant background paralleled FDG uptake results in both murine and human cases (higher uptake in KRAS/LKB1 double mutant status), thus providing a solid basis for the effectiveness of combined therapy in the different genetic statuses of the tumors. Thus, co-clinical trials harbor a strong potential in the analysis of resistance mechanisms, as well as in the effective and rapid translation of the acquired information to clinical practice.

## 3. ACQUIRED RESISTANCE 

Preclinical studies may provide a key contribution to identify molecular bases of resistance to molecular agents and to suggest novel therapeutic strategies to be tested in the clinical setting. To this purpose, several preclinical *in vitro*/*in vivo* models of acquired resistance to the targeted agents used in the clinical practice have been developed, by using two main strategies (Table **2**):

- Genetic manipulation to model genotypes of acquired resistance;

- *In vitro*/*in vivo* selection of resistant models.

### 3.1. Genetic Manipulation to Model Genotypes of Acquired Resistance 

Given the genetic instability of cancer cells with the contribution of drug-induced selective pressure, the onset of further genetic modifications such as gene amplification, deletion, or point mutations could allow them to switch to alternative survival pathways, thus inducing acquired resistance [10]. The exact contribution of cancer-associated mutations in the development of acquired resistance to targeted agents may be assessed in preclinical studies through two different cell-based approaches. 

In the first approach, the cDNA corresponding to putative oncogenic alleles is introduced into cells by transfection or viral transduction. Similarly, plasmids or viral vectors can be employed to deliver small interfering RNAs (siRNA) to tumor suppressor genes and assess the functional effects of gene inactivation by down-regulation. The introduced genetic materials (DNAs and RNAs) may exist in cells either stably or transiently. For stable transfections, introduced genetic materials that usually have a marker gene for selection (transgenes) are integrated into the host genome and sustain transgene expression even after host cells replicate. In contrast with stably transfected genes, transiently transfected genes are only expressed for a limited period of time and are not integrated into the genome. Transiently transfected genetic materials can be lost by environmental factors and cell division, so the choice of stable or transient transfection depends on the aims of the experiment. A transient transfection may be suitable for *in vitro* studies aimed to evaluate the short-term response to specific drugs. Conversely, stable transfection is mandatory in mice studies; moreover, it allows to obtain models available for multiple experiments. So far, several transfection methods have been described, to be preferred depending on cell type and research purposes: They include chemical, physical and biological (virus-mediated) methods [46] (Table **3**). Each of these method shows specific advantages and disadvantages in terms of gene delivery efficiency, handiness, reproducibility: the ideal method should have high transfection efficiency, low cell toxicity, minimal effects on normal physiology and be easy to use and reproducible [47]. 

Overall, methods based on cDNA/siRNA introduction in cancer cells are still widely used in cancer research to model genotypes of acquired resistance to targeted agents. For example, Eichorn and colleagues performed a genome wide loss-of-function short hairpin RNA screen to identify novel modulators of resistance to lapatinib, a dual EGFR/HER2 TKI approved for the treatment of metastatic breast cancers that overexpresses the HER2 receptor. In this way, they identified the tumor suppressor PTEN as a modulator of lapatinib sensitivity. In addition, they showed that two dominant activating mutations in the PI3K catalytic, alpha polypeptide (PI3K-CA), E545K and H1047R, which are prevalent in breast cancer, also confer resistance to lapatinib [48]. 

Conversely, Li *et al.* infected cells with lentiviruses expressing either wild-type kinases (Src, Fyn Lyn, EGFR and others) or kinase alleles with gatekeeper mutations. Through this approach, they showed that Src Family Kinases (SFKs), as well as EGFR, are relevant targets for the Src inhibitor dasatinib and that acquired T790M mutations render cells resistant not only to erlotinib and gefitinib, but also to dasatinib [49]. In a recent report, human glioblastoma cells have been transduced with retroviruses encoding Notch delta-like ligand 4 (DLL4), grown as tumor xenografts and then treated with the anti-VEGF mAb bevacizumab. In this way, the authors demonstrated that combination therapy to block DLL4-Notch signaling may enhance the efficacy of VEGF inhibitors, particularly in DLL4-upregulated tumors [50]. Since a large body of evidence supports the role of microRNAs (miRNAs) in cancer development and progression, as well as in the response to specific drugs [51], some reports investigated their contribution to acquired resistance to targeted agents. Stable transfections of gefitinib resistant NSCLC cells with green fluorescent protein (GFP)-lentivirus constructs containing either full-length miRNAs or anti-miRNAs demonstrated that overexpression (miR-103 and miR-203) or knock down (miR-221 and -30c) of miRNAs differentially regulated by EGFR and Met TKRs could restore gefitinib sensitivity *in vitro* and *in vivo* [52]. Although these approaches can help to study mechanisms of acquired resistance to anticancer molecular agents, they are typically hampered by at least two major caveats. First, ectopic expression is often limited by artificially high gene expression levels, due to the control of non-endogenous viral promoters, unable to recapitulate what occurs in human cancers. Consistently, ectopic overexpression of wild-type K-Ras in DiFi human colorectal cancer cells conferred resistance to cetuximab *per se*, even in absence of a specific mutation in exon 2 [53]. Otherwise, the use of siRNA may suffer from incomplete repression of targeted alleles and/or potential off-target effects. 

In the second approach, mutations are introduced into the genomes of human tumor cells using gene-targeting methods. Although more laborious than overexpression-based methods, genetic manipulation of human cells has proven valuable for a variety of purposes: to express oncogenic alleles from their endogenous promoters; to selectively delete the mutated allele of an oncogene; to knock-out gene function by exon removal; and to delete both alleles of a tumor suppressor gene [54]. To these aims, several gene targeting technologies may be used so as to engineer single or multiple oncogenic alleles in cancer cells, including adenoassociated virus (AAV)-mediated homologous recombination, plasmid-based homologous recombination, Flip-in, zinc-finger nucleases- or meganucleases-based methods. The AAV-mediated homologous recombination [55] (see Box **1**) allows to obtain, from the same cell line, different clones, each provided with a specific mutation. Moreover, these cell clones will be provided with a genetically matched ‘normal cell’, thus defining an isogenic platform in which the mutated genes are expressed under their endogenous promoters, able to closely recapitulate the lesions observed in human tumors. The isogenic knock-in models, which show a peculiar ‘oncogene addiction’ phenotype, could prove useful not only to address basic biology questions, but also to investigate the molecular players involved in the occurrence of drug inefficacy. This could eventually lead to novel opportunities for development of therapeutic intervention based on the genetic background of individual tumors and rational combinations of conventional and targeted agents designed to overcome resistance to inhibitors of single oncoproteins [54]. 

By using the isogenic models, De Roock and colleagues studied association of different K-Ras mutations with clinical outcome in chemotherapy-refractory metastatic colorectal cancers treated with cetuximab. Briefly, they demonstrated the different effects of the G12 and G13 K-Ras alleles on response to cetuximab and found a significant association between the presence of a G13D mutation and survival benefit after cetuximab treatment in metastatic colorectal cancer patients [56]. Moreover, a paper by Di Nicolantonio *et al.* demonstrated the different role of PI3K and K-Ras mutations in the response to the mammalian Target Of Rapamycin (mTOR) inhibitor everolimus. In fact, while cells knocked-in for the PIK3-CA alleles H1047R and E545K showed an increased response to everolimus, oncogenic K-Ras mutations conferred resistance to this agents [57].

### 3.2. *In Vitro/In Vivo* Selection of Resistant Models

The onset of acquired resistance in cancer patients is typically observed after a variable period from the beginning of treatment. The escape mechanisms activated by cancer cells under the drug-induced selective pressure are often very complex and not limited to a single genetic alteration. A large body of evidence suggests that resistance to anticancer targeted agents could be acquired through both the selection of cell clones with oncogenic mutations and the development of new mutations. Moreover, these events may be affected by tumor-host interactions, with tumor microenvironment significantly affecting response to anticancer drugs. The role of the different cell populations of tumor microenvironment (stromal, endothelial, inflammatory cells) has been extensively studied in the case of resistance to anti-angiogenic drugs. Data from RCC xenograft models indicate that resistance to the Vascular Endothelial Growth Factor Receptor (VEGFR) antagonist sorafenib, and most of the associated changes in gene expression, are reversed by re-implantation of the resistant xenografts into untreated mice. This prompt reversibility of resistance argues against any permanent genetic or epigenetic change in the tumor cells as an underlying mechanism. Conversely, it suggests that resistance, in part, relates to physiological changes in the microenvironment, enabling reestablishment of angiogenesis in the setting of VEGFR blockade [58]. As a confirm, Huang *et al.* used an intermittent dosing animal model to show that the development of sunitinib resistance was accompanied by evasion of sunitinib’s antiangiogenic effects and by increased expression of tumor derived IL-8 [59]. In this and other cases, genetic manipulations targeting a single gene in cancer cells may be not sufficient to recapitulate the complex scenario of the acquired resistance to targeted agents observed in cancer patients. 

**Fig. (1):**
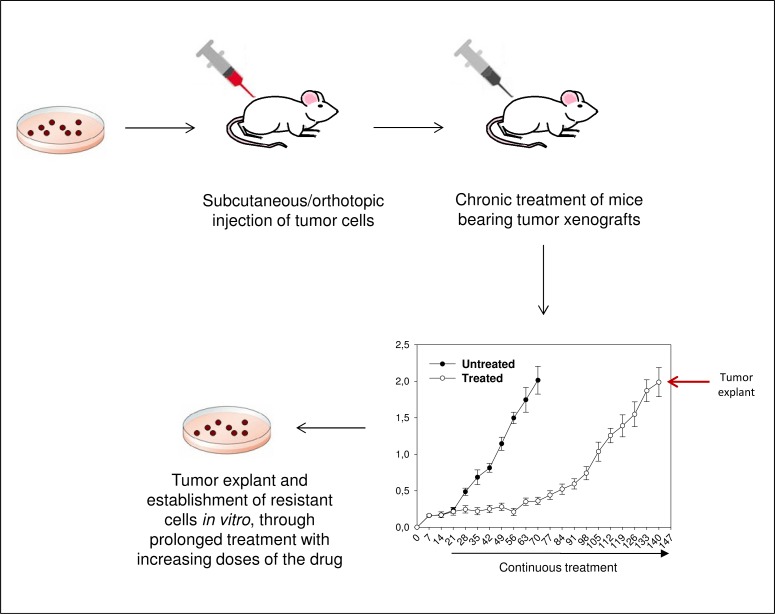
*In vivo/in vitro* selection of human cancer cells with acquired resistance to anticancer drug.

A validated protocol to reproduce the onset of acquired resistance under the drug-induced selective pressure in preclinical studies is based on an *in vivo*/*in vitro* selection of cancer cells with acquired resistance to molecular agents after a chronic treatment with the selected drug. To this purpose, human cancer cells may be xenografted in immunodeficient mice, such as athymic nude or SCID mice. The injection of human cancer cells may be made both subcutaneously (s.c.), into the dorsal flank, regardless of their native tissue type, or orthotopically, by implanting tumor cells into the organ of origin. The choice between these approaches depends on several factors, mainly the accessibility of the organ for the implantation and the subsequent removal of the tumor. After the injection of tumor cells, when established xenografts become palpable with a tumor size of ~0.2–0.3 cm^3^, mice are addressed to receive continuous treatment with the selected drugs, at clinically relevant doses. Chronic treatment with anticancer molecular agents typically cause a strong tumor inhibition. However, after a variable period of continuous treatment (10 to 30 weeks), tumors begin to grow, eventually reaching a growth rate comparable with untreated control tumors. Therefore, mice are sacrificed, tumors excised and the derived cells established *in vitro* through prolonged treatment with increasing doses of the drug (Fig. **1**). After having controlled morphological appearance, *in vitro* growth rate, and soft-agar cloning efficiency of the harvested cells compared to that of parental cells, a derived cell line with acquired resistance to the selected drug is available to study mechanisms of resistance both *in vitro* and *in vivo* [60]. Based on this protocol, Ciardiello *et al.* generated Cetuximab Resistant (CR) and Gefitinib Resistant (GR) cell lines derived from the cetuximab sensitive, GEO human colorectal cancer cells. These and other cells have been proven to be very useful tools to study different mechanisms of acquired resistance to targeted agents, possibly coexisting in the same cell line. For example, we demonstrated that VEGFR-1 may contribute to acquired resistance to EGFR inhibitors in colorectal and breast cancer models by activating the PI3K/Akt pathway [61]. More recently, we also demonstrated that cross-talks between Sphingosine Kinase 1 (SphK1) and EGFR-dependent signaling pathways could mediate resistance to cetuximab in colorectal cancers [62]. Following the same approach, Carbone and colleagues established and validated two *in vivo* pancreatic cancer models with evasive resistance to anti-VEGF mAb bevacizumab, PANC-1-BR (Bevacizumab Resistant) and COLO357FG-BR. They identified several pro-inflammatory factors responsible for increased aggressiveness of bevacizumab-resistant pancreatic tumors that acted both in a paracrine manner to stimulate the recruitment of CD11b+ pro-angiogenic myeloid cells and in an autocrine manner to induce epithelial- to- mesenchymal transition (EMT) [63]. Two recent papers described breast cancer cell models of acquired resistance to the HER2-targeting agents trastuzumab and lapatinib. These models were generated by maintaining SK-BR-3 and BT474 human breast cancer cells in gradually increasing concentrations of trastuzumab or lapatinib. In both articles, Src TK activation was shown to be an important mechanism of resistance to HER2 inhibitors, suggesting the potential clinical application of the combined HER2/Src targeting early in the treatment of HER2 positive breast cancers, in order to prevent or overcome resistance to HER2 inhibitors [64, 65]. Similarly, Yano *et al.* first discovered a novel mechanism of acquired resistance to gefitinib in EGFR-mutant human NSCLC cells PC-9 and HCC827. The induction of resistance seemed to be mediated by Hepatocyte Growth Factor (HGF)-mediated activation of the PI3K/Akt signaling pathway via phosphorylation of Met [66]. Acquired resistance to Anaplastic lymphoma kinase (ALK) TKIs such as crizotinib has been described in patients with NSCLC positive for the echinoderm microtubule-associated protein–like 4 (EML4)–ALK fusion protein. Recently, Tanizaki and colleagues identified the EGF-mediated activation of HER family signaling as a mechanism driving such resistance by establishing lines of EML4-ALK–positive H3122 lung cancer cells resistant to the ALK inhibitor TAE684 (H3122/TR cells) [67]. In the field of hematological malignancies, different mechanisms of acquired resistance to imatinib in chronic myelogenous leukemia were clarified by using K562-Imatinib Resistant (IR) cells generated through serial prolonged exposures to increasing concentrations of imatinib (from 1 nM to 1 μM) [68-70].

Several reports suggested that the combined *in vivo*/*in vitro* selection could be a more reliable technique compared to the plain *in vitro* exposure of cancer cells to increasing doses of the drugs. In fact, in most cases, only the double selection protocol allows to obtain cells that retain a resistant phenotype even after several culture passages [61]. Importantly, the generation of such models could help to unveil novel mechanisms of acquired resistance to anticancer targeted agents. In fact, they are suitable for high-throughput genomic or proteomic screenings able to identify molecular alterations arisen in resistant cells compared to their sensitive counterparts. Recently, by using a proteomic approach based on combined 2D-DIGE and MS analysis, we found that increased anaerobic metabolism is a distinctive signature in colorectal cancer cellular models of resistance to cetuximab [71].

A feasible and innovative model for studying mechanisms of resistance to anticancer molecular agents could be the use of a series of human cancer specimens directly transplanted into mice, in order to generate study populations that could be concomitantly profiled for biomarker assessment and randomized for prospective treatment with targeted agents. This strategy could potentially overcome the limitations of immortalized cancer cells which are commonly employed in *in vitro* and *in vivo* preclinical experiments. These cell lines have been indeed adapted to grow on plastic in the laboratory for decades and thus exhibit a genetic background, a biological compliance and phenotypic features different from original cancers in patients. In fact, while each cancer in each individual is a separate entity, with a unique natural history and a number of unpredictable patient-specific interacting events, cancer cell lines used in preclinical research have to be considered only representative ‘cases’. Therefore, experiments with cell lines cannot recapitulate the wide heterogeneity of human malignancy that occurs among individuals within a certain population. The absence of genetic heterogeneity, or at least a strong tendency towards an artificially uniform tumor evolution, is also a limitation of genetically defined mouse models of cancer, which usually develop stereotypical lesions triggered by the same initiating oncogenic hit [72]. In order to create a platform of patient-derived xenografts (‘xenopatients’), a biobank of surgical materials stored under viable conditions is required. Following surgical removal from patients, each specimen is cut in small pieces and different fragments are implanted in different mice. After engraftment and tumor mass formation, the tumors are passaged and serially expanded for two generations until production of independent xenograft lines from the same patient tumor. By combining the use of severely immunocompromised NOD/SCID animals with optimization of patient-to-mouse transfer procedures, it is possible to achieve a large percentage of successful engraftments. When Bertotti and colleagues applied this strategy to a cohort from 85 patient-derived, genetically characterized metastatic colorectal cancer samples, they obtained several lines of evidence supporting its robustness and predictive power. In fact, despite their ectopic (s.c.) site of growth, colorectal xenografts retained the morphological characteristics of the corresponding patient’s lesion. Moreover, no critical sampling bias in the random selection of cancer fragments for implantation was found, and serial mouse passaging seemed not grossly alter the genetic makeup of tumors, at least when considering global copy number changes and hotspot oncogenic mutations. More importantly, the frequency of tumor regression, disease stabilization and disease progression following cetuximab treatment was in line with the clinical data reported in humans; and, identical to clinical observations, K-Ras (codon 12 and 13) mutant xenografts were all resistant to EGFR blockade by cetuximab. Therefore, this preclinical platform may provide an instructive framework for additional biomarker discovery, for generation of predictive classifiers aimed to a better patient stratification, and for testing novel investigational therapies. For example, analysis of the metastatic colorectal cancer xenopatients cohort allowed to identify HER2 as a predictor of resistance to anti-EGFR antibodies and as a predictor of response to combinatorial therapies against HER2 and EGFR in this tumor setting [72].

## Figures and Tables

**Table 1.** *In Vitro* and *In Vivo* Models for Analysis of Intrinsic Resistance to Anticancer Biological Agents

Cellular models				Animal models			
Human immortalized cancer cell lines derived from cancer patients showing primary resistance				Human tumor xenografts in mice			
Primary cultures of cells often directly obtained at the time of diagnosis from human cancers				Syngeneic mouse models			
				Genetically engineered mouse models (GEMMs)			
**Clinical settings **	**Drug resistance **	**Models **	**References **	**Clinical settings **	**Drug resistance **	**Models **	**References **
Breast Cancer NSCLC Colorectal Cancer Colorectal Cancer Colorectal Cancer Mantle Cell Lymphoma	Trastuzumab Erlotinib-Gefitinib Cetuximab-Panitumumab Vemurafenib Selumetinib Bortezomib	JIMT-1 KPL-4 A549 H460 H1299 GLC-82 SW480 LS174T HCT116 LoVo VAC0432 SNU-C5 HT29 KM20 WiDr HCA7 CaCo2 COLO320DM RKO CO115 DLD-1 SW837 MINO REC1	Tanner *et al.*, 2004 Kurebayashi *et al.*, 1999. Orzaez *et al.*, 2012; Milligan *et al.*, 2009; Gorzalczany *et al.*, 2011. Rosa *et al.*, 2011. Bollag *et al.*, 2010 Yeh *et al.*, 2007 Balmanno *et al.*, 2009.	Lewis Lung Carcinoma Lymphoma Pancreatic Neuroendocrine Carcinoma Lung Cancer	Anti-VEGF mAb Anti-VEGF mAb Anti.IGF-1R Docetaxel+ Selumetinib	LLC tumor EL4 tumor RIP1-Tag2 transgenic mouse model GEMM models	Shojaei *et al.*, 2007; Shojaei *et al.,* 2008. Ulanet *et al.*, 2010. Chen *et al.*, 2012

**Table 2.** In Vitro and In Vivo Models for Analysis of Acquired Resistance to Anticancer Biological Agents

Genetic manipulation to model genotypes of acquired resistance				*In vitro/in vivo* selection of resistant models			
Methods based on cDNA/siRNA transfection				*In vivo/in vitro* treatment with the selected drug			
Gene-targeting methods				Treatment of patient-derived xenografts (‘xenopatients’) in mice			
**Clinical settings **	**Drug resistance **	**Models **	**References **	**Clinical settings **	**Drug resistance **	**Models **	**References **
Breast Cancer Lung Cancer Glioblastoma NSCLC Colorectal cancer	Lapatinib Dasatinib Bevacizumab Gefitinib Cetuximab Everolimus	Genome wide loss-of-function short hairpin RNA screen Expression of wild-type kinases or mutated kinase alleles Expression of DLL-4 Full-length miRNA or anti-miRNAs Isogenic knock-in model Isogenic knock-in model	Eichhorn *et al.*, 2008. Li *et al.*, 2010. Li *et al.*, 2011. Garofali *et al.*, 2011. De Roock *et al.*, 2010. Di Nicolantonio *et al.*, 2010.	Pancreatic Cancer Breast Cancer NSCLC Lung Cancer Chronic Myelogenous Leukemia Colorectal Cancer	Bevacizumab Trastuzumab Lapatinib Gefitinib TAE684 Imatinib Cetuximab Cetuximab-Panitumumab	PANC-1-BR and COLO357FG-BR *in vivo* models SK-BR-3 cells BT474 cells PC-9 and HCC827 cells H3122/TR cells K562-IR GEO-CR Xenopatients	Carbone *et al.*, 2011. Rexer *et al.*, 2011. Zhang *et al.*, 2011. Donev *et al.*, 2011. Tanizaki *et al*., 2012. Arunasree *et al.*, 2008; Marfe *et al.*, 2011; Liu *et al.*, 2012. Monteleone *et al.*, 2012. Bertotti *et al.*, 2011.

**Table 3.** Transfection Methods

Chemical methods	Physical methods	Biological methods (transduction)
Cationic polymers	Microinjection	Adenovirus
Calcium phosphate	Biolistic particle delivery	Adenoassoiated virus
Cationic lipids	Electroporation	Herpes Simplex virus
Cationic amino acids	Laser-based transfection	

**Box 1.** Gene targeting technologies: adenoassociated virus (AAV)- mediated homologous recombination.

In the AAV-mediated protocol, the homologous recombination cassette is cloned within the AAV inverted terminal repeats. The cassette consists of two “homology arms”, sequences of about 1 kb, one of which contains the specific mutation. A selectable marker, such as the Neomycin Resistance gene (Neo), is placed between the homology arms flanked by two LoxP sites; since Cre recombinase catalyzes site-specific recombination between LoxP sites, this architecture allows the excision of the Neo cassette from the genome of the targeted cells and the possibility of recycling the resistance marker for the sequential introduction of multiple alleles in the same cell. After infection with recombinant AAV (rAAV) and selection with the selective antibiotic G418, clones with locus-specific integration of the targeted alleles could be identified through a PCR screening approach. Positive clones are expanded and genomic DNA and RNA are extracted to sequence the targeted region, in order to independently confirm the presence and the expression of the specific mutations.

## ABBREVIATIONS

ALK: = Anaplastic lymphoma kinase
APL: = Acute promyeolocytic leukemia
CRC: = Colorectal cancer
EMT: = Epithelial to mesenchimal transition
GEMM: = Genetically engineered mouse model
mAb: = Monoclonal antibody
NK: = Natural killer
NSCLC: = Non-small cell lung cancer
TKI: = Tyrosine kinase inhibitor
VEGF: = Vascular endothelial growth factor
